# Phenylbutyrate—a pan-HDAC inhibitor—suppresses proliferation of glioblastoma LN-229 cell line

**DOI:** 10.1007/s13277-015-3781-8

**Published:** 2015-08-11

**Authors:** Magdalena Kusaczuk, Rafał Krętowski, Marek Bartoszewicz, Marzanna Cechowska-Pasko

**Affiliations:** 1 Department of Pharmaceutical Biochemistry, Medical University of Białystok, Mickiewicza 2A, 15-222 Białystok, Poland; 2 Department of Microbiology, Institute of Biology, University of Białystok, Białystok, Poland

**Keywords:** Apoptosis, Cell cycle, Glioblastoma, Histone deacetylase inhibitors, Phenylbutyrate

## Abstract

Phenylbutyrate (PBA) is a histone deacetylase inhibitor known for inducing differentiation, cell cycle arrest, and apoptosis in various cancer cells. However, the effects of PBA seem to be very cell-type-specific and sometimes limited exclusively to a particular cell line. Here, we provided novel information concerning cellular effects of PBA in LN-229 and LN-18 glioblastoma cell lines which have not been previously evaluated in context of PBA exposure. We found that LN-18 cells were PBA-insensitive even at high concentrations of PBA. In contrary, in LN-229 cells, 5 and 15 mmol/L PBA inhibited cell growth and proliferation mainly by causing prominent changes in cell morphology and promoting S- and G2/M-dependent cell cycle arrest. Moreover, we observed nearly a 3-fold increase in apoptosis of LN-229 cells treated with 15 mmol/L PBA, in comparison to control. Furthermore, PBA was found to up-regulate the expression of *p21* whereas *p53* expression level remained unchanged. We also showed that PBA down-regulated the expression of the anti-apoptotic genes *Bcl-2*/*Bcl-X*_*L*_, however without affecting the expression of pro-apoptotic *Bax* and *Bim*. Taken together, our results suggest that PBA might potentially be considered as an agent slowing-down the progress of glioblastoma; however, further analyses are still needed to comprehensively resolve the nature of its activity in this type of cancer.

## Introduction

Despite the extensive efforts undertaken to treat glioblastoma, this cancer is now considered to be the most frequent type of glial tumors with poor prognosis of survival. Current approaches of treatment include radiotherapy and chemotherapy as well as surgical interventions, any of which is effective enough to treat this incurable malignancy. The probable reason of the difficulties in developing effective therapy against glioblastoma is connected with the fact that these cancer cells are very anaplastic and heterogeneous and highly active in migrating along fiber tract and blood vessels to invade the brain [1].

Recently, it has become evident that cancer development is basically regulated by both genetic as well as epigenetic alterations. Genetic changes in malignant gliomas have been extensively investigated and include mutations in main tumor suppressor genes such as *p53* or *PTEN* [2, 3] and deletions of some parts of the chromosomes (e.g., 6q26-27, 1p36.23, 17p13.3-12) [4]. Currently, a great deal of attention has also been shifted toward epigenetic regulation of cancer genesis and progression. Methylation of the CpG islands in the promoter regions of genes and chromatin structure remodeling have also been identified as an important processes involved in tumor development [5]. Alterations of the chromatin architecture are regulated by histone acetylation/deacetylation modifications [6]. Nucleosomes composed of histones showing low levels of acetylation are the hallmark of transcriptionally silent chromatin; reversely, relaxed chromatin structure is composed of highly acetylated histones [7, 8]. Histone acetylation status is guarded by two crucial groups of counteracting enzymes: histone acetyltransferases (HATs) and histone deacetylases (HDACs) [7, 8]. HATs transfer acetyl groups from acetyl-coenzyme A onto the amino groups of lysine residues of histones, resulting in transcriptional activation. In contrary, HDACs catalyze the removal of these acetyl moieties from histone proteins causing chromatin tightening and transcriptional repression [7, 9].

Acetylation homeostasis can be modulated by the group of compounds called the histone deacetylase inhibitors (HDACIs). Yet, five classes of HDACIs have been distinguished according to their structural characteristics: (i) organic hydroxamic acids, (ii) short-chain fatty acids, (iii) benzamides, (iv) cyclic tetrapeptides, and (v) sulfonamide anilides [6, 7, 10].

Phenylbutyric acid (PBA) is a short-chain fatty acid known to possess broad spectrum of molecular functions. It has been primarily developed as an ammonia scavenger in urea cycle disorder treatment. However, multiple researches conducted over years have demonstrated other biological activities of PBA. In this regard, PBA has been shown to display the activity of a chemical chaperone at high concentrations and to possess the ability of inhibiting HDACs [7]. PBA is characterized by good bioavailability in vivo of approximately 3 mmol/L; nevertheless, higher concentrations ranging between 1 and 5 mmol/L have also been stated [11–13]. Because of the low cytotoxicity of PBA and the effective cerebrospinal fluid penetration, an interesting area of investigation concerning its utility in brain tumor research has been opened [14].

Among various activities of PBA, it has been demonstrated to be the reversible inhibitor of class I and II HDACs [10]. PBA mode of action in cancer cells has been attributed to reduced proliferation [15], enhanced differentiation [1, 16], increased apoptosis [1, 17, 18], and cell cycle arrest [14, 18]. However, the molecular pathways underlying these processes still seem to be only partially discovered. Apoptosis evoked by PBA treatment has been suggested to be linked to the down-regulation of many anti-apoptotic genes such as *Bcl-X*_*L*_, *Bcl-2*, or survivin, as well as disrupted signal transduction pathways responsible for cell survival, including down-regulation of the and Akt and nuclear factor-κB (NF-κB) signaling [19–21]. Moreover, the expression of some pro-apoptotic (e.g., Bid, caspase-3, caspase-7, and caspase-8) molecules has been significantly up-regulated after PBA supplementation [22, 23]. It has also been widely demonstrated that the pro-apoptotic and cell cycle inhibiting effects of butyrates are likely to be mediated by restoration of the p53-dependent signaling pathways [24–26]. Furthermore, it has been proven that cell cycle arrest is frequently connected with the strong induction of the P21 expression [15, 27]. Other cell cycle regulatory proteins have also been found to be deregulated after PBA treatment. The expressions of both cyclins and cyclin-dependent kinase (Cdk) inhibitors such as Cdk2, Cdk4, cyclin D1, or cyclin A have been shown to be modulated by PBA [28–30]. Nevertheless, cellular responses and gene expression patterns initiated after PBA treatment are not universal and seem to be very cell-type-specific. Although a great deal of research concerning PBA functioning in cancer has already been undertaken, the mechanism of phenylbutyrate action in glioblastoma cells has not been comprehensively solved yet.

To extend the range of knowledge about PBA influence on glioblastoma cells, we made an effort to determine the cell-type-specific effects of phenylbutyrate in LN-18 and LN-229 cell lines. PBA was found to have HDAC inhibitory effect in both cell lines; however, only LN-229 cells displayed growth inhibitory effect after PBA treatment. Our data indicate that LN-229 cells showed marked dose-dependent inhibition of cell proliferation, visible changes in cell phenotype, and increased apoptosis after incubation with high concentration of PBA in growth medium. Moreover, we observed pronounced alterations in the cell cycle distribution, with noticeable tendency to arrest the cells in S and G2/M phases. These effects were accompanied by enhanced expression of *p21* transcript, while the unchanged *p53* expression status was observed, suggesting p53-independent mode of action. Furthermore, the expressions of the main anti-apoptotic genes *Bcl-2*/*Bcl-X*_*L*_ were significantly down-regulated. To our knowledge, this is the first attempt to evaluate the effect of PBA on glioblastoma LN-229 cells.

## Materials and methods

### Reagents

Dulbecco’s modified Eagle’s medium (DMEM), containing glucose at 4.5 mg/mL (25 mM) with Glutamax, penicillin, streptomycin, trypsin-EDTA, and High Capacity RNA-to-cDNA Kit were provided by Invitrogen (San Diego, USA); passive lysis buffer, ReliaPrep RNA Cell Miniprep System, and HDAC-Glo™ I/II Assay and Screening System by Promega (Madison, USA); FBS Gold by Gibco (USA); fluorescein isothiocyanate (FITC) Annexin V Apoptosis Detection Kit I by BD Pharmingen (CA, USA); and RNase by AppliChem (Darmstadt, Germany). 4-Phenylbutyrate was purchased from Enzo Life Sciences, Inc. (Lausen, Switzerland) and molecular-grade purity water from Sigma-Aldrich (St. Louis, MO, USA),

### Cell cultures

Human glioblastoma cell lines LN-229 and LN-18 were kindly provided by Prof. Cezary Marcinkiewicz from the Department of Neuroscience, Temple University, Philadelphia, USA. Cells were maintained in high-glucose DMEM supplemented with 5 % heat-inactivated fetal bovine serum GOLD (FBS GOLD), 2 mmol/L l-glutamine, penicillin (100 U/mL), and streptomycin (100 μg/mL). Cells were cultured in Falcon flasks (BD) in a 5 % CO_2_ incubator (Galaxy S+; New Brunswick), at 37 °C. Subconfluent cultures were detached with 0.05 % trypsin 0.02 % EDTA in calcium-free phosphate-buffered saline (PBS) and counted in cell counter Scepter (Millipore).

### Determination of HDAC inhibitor potency

HDAC activity was measured using luminescent HDAC-Glo™ I/II Assay and Screening System (Promega) according to the manufacturer’s protocol. Briefly, cells were seeded in 96-well white-walled culture plates at a density of 10,000 cells/well. Attached cells were incubated with various concentrations of PBA solution (0.5–15 mmol/L) for 1 h at 37 °C. After incubation period, 100 μL of the HDAC-Glo™ I/II Reagent with Triton® X-100 in a final concentration of 1 % was added to each well, and cells were incubated at room temperature for another 35 min. Luminescence was measured on a microplate reader (Tecan, Switzerland), at signal steady-state 35 min after adding the HDAC-Glo™ I/II Reagent to the cells. Experiment was performed in triplicates.

### Cell viability

Cell viability was measured according to the method of Carmichael et al. [31] using 3-(4,5-dimethylthiazol-2-yl)-2,5-diphenyltetrazolium bromide (MTT). Briefly, cells were seeded in 24-well plate at a density of 5 × 10^4^ per well. Confluent cells, cultured for 24 and 48 h with different concentrations of PBA (0.5–15 mmol/L), were washed three times with PBS and then incubated with 1 mL of MTT solution (0.25 mg/mL in PBS) for 4 h at 37 °C in 5 % CO_2_ in an incubator. The medium was removed, and 1 mL of 0.1 mmol/L HCl in absolute isopropanol was added. Absorbance of converted dye in living cells was measured at wavelength of 570 nm on a microplate reader (Tecan). The viability of LN-229 and LN-18 cells was calculated on the basis of optical density, as a percentage of the cell survival in PBA-treated wells in relation to cell survival in the control wells. All the experiments were done in triplicates in at least three cultures.

### Cell morphological analysis

Acridine orange (AO)/ethidium bromide (EtB) double staining and fluorescent as well as phase contrast microscopy was used to observe morphological changes in the glioblastoma LN-229 cells. AO is taken up by both viable and dead cells. Green fluorescence occurs when AO binds to double-stranded DNA in living cells and red fluorescence, when it binds to single-stranded DNA which dominates in dead cells. EtB is excluded from living cells [32]. Of LN-229 cells, 2.5 × 10^5^ were seeded into six-well plates and incubated with 5 or 15 mmol/L PBA for 24 and 48 h at 37 °C in a humidified, 5 % CO_2_ atmosphere. After incubation, the cells were stained with mixture of AO (10 μmol/L) and EtB (10 μmol/L). The cells were viewed under a fluorescent and phase contrast microscope (Olympus, CKX 41) at × 100 or × 200 magnification.

### Detection of apoptosis

The cells (2.5 × 10^5^ in 2 mL of medium) were seeded in six-well plates and incubated until they achieved confluence. The LN-229 cells were incubated in the high-glucose DMEM with 5 or 15 mmol/L PBA. The incubation was continued for 24 and 48 h. The cells were trypsinized and resuspended in DMEM and then in a binding buffer. Cells were stained with FITC Annexin V and PI for 15 min at room temperature in the dark following the manufacturer’s instructions (FITC Annexin V apoptosis detection Kit I). Flow cytometry was performed using the FACSCanto II cytometer (Becton Dickinson). Data were analyzed with FACSDiva software, and dead cells were excluded based on forward- and side-scatter parameters. Percentage of apoptotic cells as a sum of Q2 and Q4 quadrant and necrotic cells as a Q1 quadrant population of analyzed cells was presented.

### Cell cycle analysis

LN-229 cells were seeded into six-well plates at a density of 2.5 × 10^5^ cells per well and incubated for 24 and 48 h with 5 or 15 mmol/L PBA. After this time, cells were detached and fixed with 1 mL of 70 % ethanol and kept at −20 °C for at least one night. Subsequently, the cells were washed, resuspended in PBS, and treated with 50 mg/mL of DNase-free RNase A (AppliChem). Finally, cells were stained with 100 μg/mL of PI and fluorescence was read by a flow cytometer FACSCanto II.

### RNA isolation

Total cellular RNA was extracted using the ReliaPrep RNA Cell Miniprep System (Promega) and treated with DNase I according to the manufacturer’s protocol. Quantity of the purified RNA was measured spectrophotometrically (A260/A280) using NanoPhotometer (Implen, Germany). Quality of isolated RNA was assessed by capillary electrophoretic measurement of RNA integrity number (RIN) using Bioanalyzer 2100 (Agilent, Palo Alto, CA). Samples with RIN values exceeding 9 were taken for further analyses.

### Gene expression analysis

Reverse transcriptase (RT) reactions were performed using a High Capacity RNA-to-cDNA Kit (Invitrogen) according to the manufacturer’s protocol. Briefly, 1 μg of purified total RNA was used in a 20-μL reaction mixture containing dNTPs, random octamers, and oligo dT-16 primers and MuLV RT. For real-time qPCR reactions, 2 μL of cDNA served as a template to subsequent product amplification using 2xHS-PCR Master Mix SYBR A (A&A Biotechnology, Poland). The sequences of the PCR primers were as previously described: *p53*: F-GTTCCGAGAGCTGAATGAGG, R-TTATGGCGGGAGGTAGACTG [33], *p21*: F- GGAAGACCATGTGGACCTGT, R-GGCGTTTGGAGTGGTAGAAA [33],*Bax*: F-GAGAGGTCTTTTTCCGAGTGG, R-CCTTGAGCACCAGTTTGCTG [34],*Bim*: F-CGTTAAACTCGTCTCCAATACGC, R-CGTTAAACTCGTCTCCAATACGC [35],*Bcl-2*: F- CTGCACCTGACGCCCTTCACC, R-CACATGACCCCACCGAACTCAAAGA [36],*Bcl-X*_*L*_: F-GATCCCCATGGCAGCAGTAAAGCAAG, R-CCCCATCCCGGAAGAGTTCATTCACT [36], *TRAIL*: F-GCTCTGGGCCGCAAAAT, R-TGCAAGTTGCTCAGGAATGAA [37] and for housekeeping *RPL13A*: F-CTATGACCAATAGGAAGAGCAACC, R-GCAGAGTATATGACCAGGTGGAA [38]. Primers were additionally checked for their accuracy by BLAST and Primer-BLAST software. Reaction parameters were as follows: initial denaturation at 95 °C for 3 min, followed by 40 cycles of 95 °C for 1 min, 59 to 69 °C for 30 s, and 72 °C for 45 s. Real-time qPCR reactions were run in triplicates in CFX Connect Real-Time PCR System (Bio-Rad), and collected data were analyzed using relative quantification method modified by Pfaffl [39].

### Statistical analysis

All data are expressed as means ± SD from three independent experiments run in triplicates. Statistical analyses were performed using Statistica Data Miner (StatSoft, Poland). The differences in each parameter were evaluated by a one-way analysis of variance (ANOVA) followed by the post hoc Tukey test for pairwise comparison between different groups. GraphPad Prism 5 software (GraphPad Software, Inc., USA) was used to count the IC_50_ values. The significant differences of means were determined at the level of *p < 0.05 and **p < 0.001.

## Results

### The effect of phenylbutyrate on HDAC activity

**Fig. 1:**
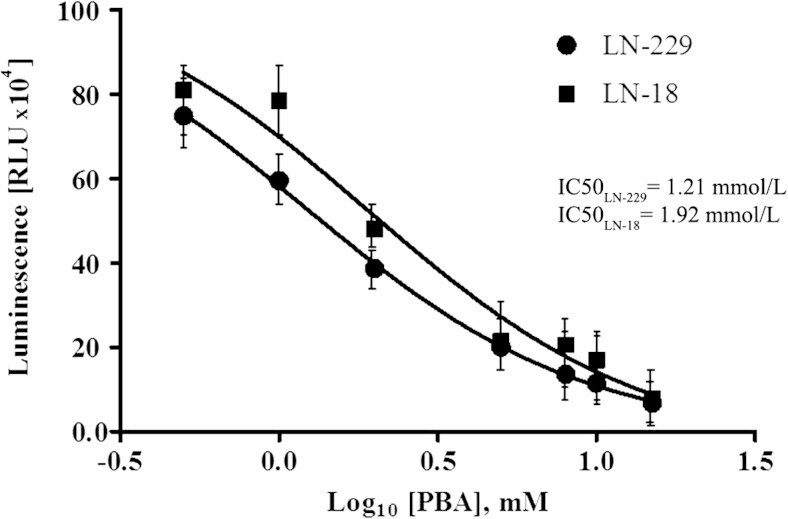
HDAC inhibitor potency of PBA. Data were plotted against logarithmic values of PBA concentrations (ranging from 0.5 to 15 mmol/L), and IC_50_ values of HDAC inhibition were determined using GraphPad Prism 5 software. Data represent the mean ± standard deviations of three replicates

To confirm HDAC inhibitory potential of PBA in glioblastoma cells, the luminescent assay using HDAC-Glo™ I/II Assay and Screening System (Promega) was performed. The relative activity of HDAC class I and II enzymes in cells subjected to phenylbutyrate treatment was measured. Figure 1 shows that PBA is very effective in inhibiting HDAC activity in both LN-229 as well as LN-18 cells and that this inhibition is strictly dose-dependent. Data were plotted against logarithmic values of PBA concentrations (from 0.5 to 15 mmol/L), and IC_50_ for HDAC inhibition was determined using GraphPad Prism 5 software. The estimated IC_50_ values for LN-229 and LN-18 cells were 1.21 and 1.92 mmol/L, respectively. These results show that PBA is slightly more potent in inhibiting HDAC activity in LN-229; however, this difference was not of statistical significance.

### The effect of phenylbutyrate on cell viability

**Fig. 2:**
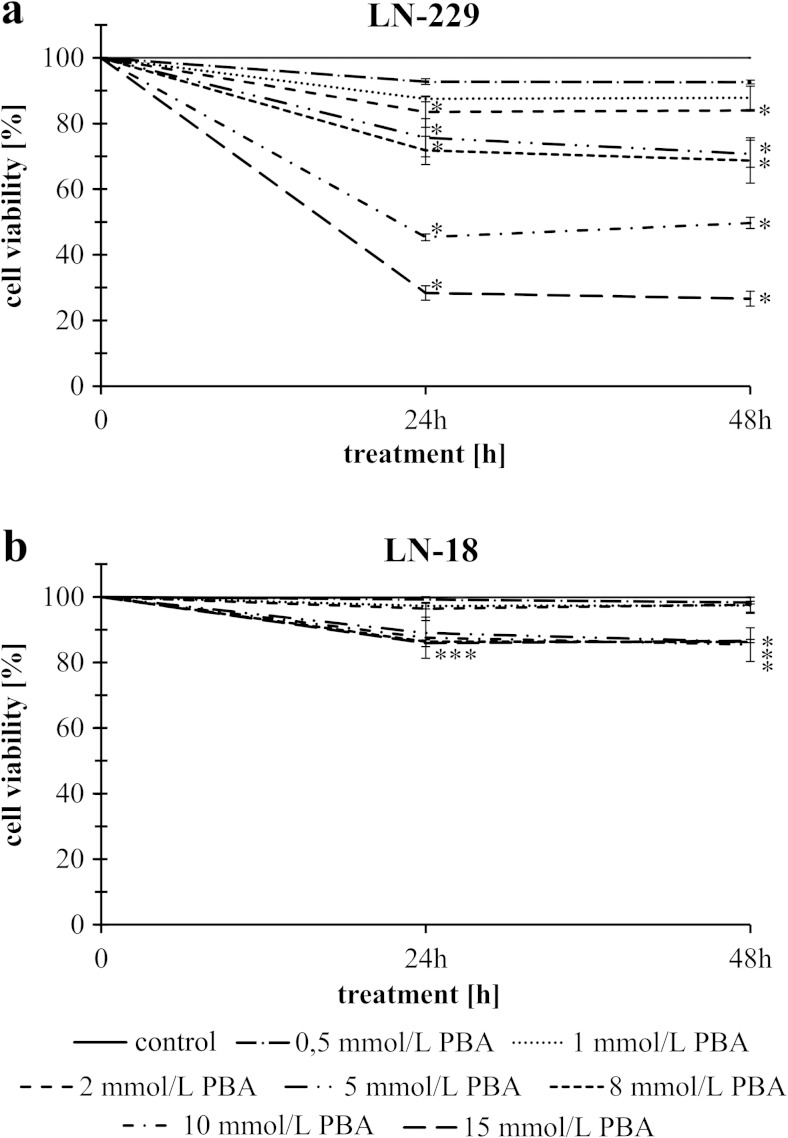
The viability of glioblastoma cancer cells treated with different concentrations of phenylbutyrate for 24 and 48 h of LN-229 (**a**) and LN-18 (**b**) cell line. The results represent means for pooled triplicate values from three independent experiments. Significant changes are expressed relative to controls and marked with *asterisks*. Statistical significance was considered if *p < 0.05

The anti-proliferative effect of PBA was assessed by MTT method in LN-229 and LN-18 glioblastoma cell lines cultured with increasing concentrations of phenylbutyrate for periods of 24 and 48 h. Figure 2a shows that PBA, in the concentrations from 0.5 to 15 mmol/L, caused dose-dependent reduction of LN-229 cell viability but notwithstanding the time. An evident inhibition of LN-229 cell viability was observed as early as 24 h after PBA treatment. In cells treated with 15 mmol/L PBA, the effect on cell viability was markedly more pronounced than in others; thereby, this concentration of PBA was found to diminish cell viability by over 70 % (Fig. 2a). The IC_50_ values of PBA were calculated using GraphPad Prism 5. For LN-229 cells, the IC_50_ value was approximately 5.45 mmol/L, while LN-18 cells were clearly PBA-insensitive. Interestingly, PBA treatment had almost no cytotoxic effect in LN-18 cell line, where the highest dose of PBA caused only 15 % reduction in cell viability (Fig. 2b). In this respect, only LN-229 cells were chosen for further study and two representative concentrations of phenylbutyrate lower (5 mmol/L) and higher (15 mmol/L) than IC_50_ value were selected for further examinations.

### The effect of phenylbutyrate on cell morphology

**Fig. 3:**
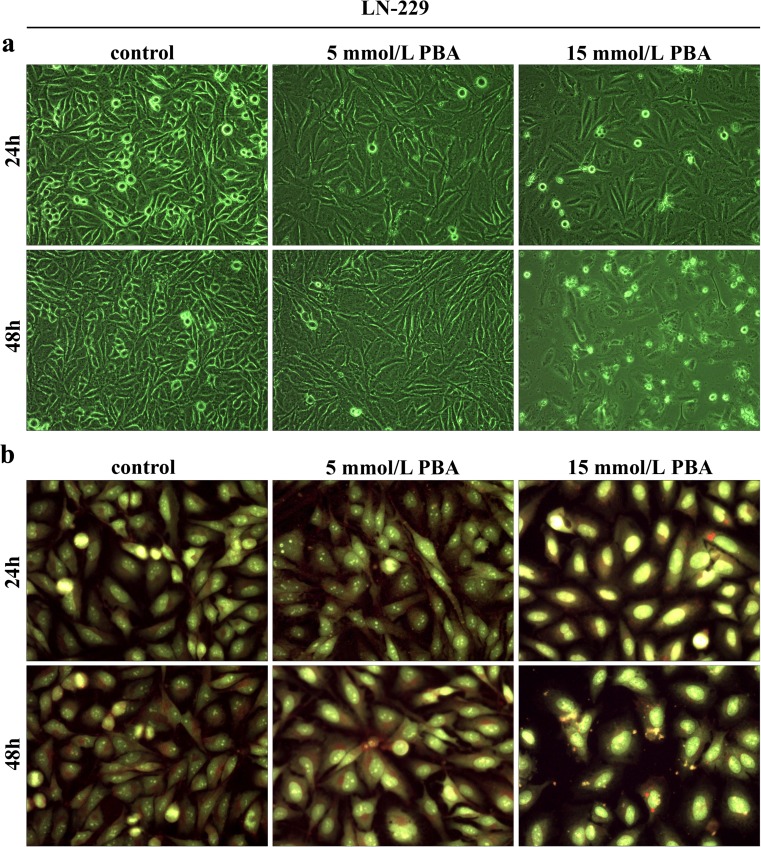
The phenotypic characteristic of LN-229 cells after 24 and 48 h of PBA treatment. Representative photographs are shown. Morphological effects induced by 5 and 15 mmol/L PBA after 24- and 48-h treatment, **a** evaluated by phase contrast microscopy (magnification × 100). Evident reduction of cell density was observed, **b** evaluated by the acridine orange/ethidium bromide staining, shown by fluorescence microscopy (magnification × 200). Markedly enlarged cells with bright yellow, condensed nuclei are visible

To check if the decrease in cell viability of LN-229 cells was mirrored in changed cell morphology and enhanced apoptosis, the microscopic observations were made. Figure 3a shows the phenotypic characteristics of LN-229 cells seen under phase contrast microscope. LN-229 cell density was visibly reduced following 24 h of PBA treatment with 5 as well as 15 mmol/L PBA; however, the most prominent proliferation-suppressive effect was observed after 48-h incubation with 15 mmol/L PBA. Moreover, the substantial changes in cell shape were also noticed (Fig. 3a). To identify the apoptotic cells, the AO/EtB staining was performed and cells were observed under fluorescent microscope. This method distinguished viable cells by appearing with green nuclei, while dead cells by showing red nuclei. Figure 3b displays representative photographs of AO/EtB-stained LN-229 cells incubated for 24 and 48 h with 5 or 15 mmol/L phenylbutyrate, in comparison to the non-treated controls. PBA treatment caused visible changes in cell morphology and density; however, we observed only slight proportion of cells with red-stained nuclei indicative of late apoptosis, which suggested that apoptotic events might be only marginally responsible for the reduction of cell density. Variations in cell morphology were PBA concentration-dependent. The most prominent effect was noticeable after 48 h of treatment with the highest concentration of phenylbutyrate. These cells showed an enlarged, swollen cell morphology and increased cytoplasm with visibly more condensed nuclei and an evident loss of spindle-shaped cells, when compared with controls.

### The effect of phenylbutyrate on apoptosis

**Fig. 4:**
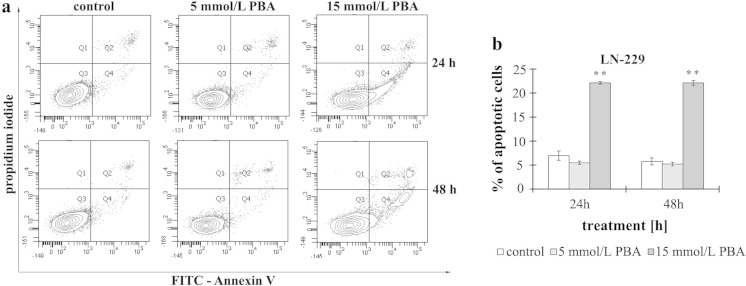
The effect of phenylbutyrate on apoptosis of glioblastoma cells. The cells were incubated with 5 and 15 mmol/L PBA for 24 and 48 h. **a** Representative FACS data for LN-229 cells subjected to Annexin V-FITC/propidium iodide staining. **b** Bar graph presenting the percentage of apoptotic cells. Mean values from three independent experiments ± SD are shown. Significant alterations are expressed relative to controls and marked with *asterisks*. Statistical significance was *p < 0.05 or **p < 0.001

To examine the mechanism of the cytostatic effect of PBA in LN-229 cells, we investigated whether the activity of phenylbutyrate is associated with apoptosis. To explicitly confirm the microscopic results of the AO/EB staining, LN-229 cells were exposed to PBA at the determined concentrations, and apoptotic cells were evaluated by flow cytometry analysis. The percent of apoptotic LN-229 cells in cultures incubated with 5 or 15 mmol/L of PBA for 24 and 48 h is reflected in Fig. 4. We did not observe any effect of phenylbutyrate on the apoptosis of LN-229 cells incubated with 5 mmol/L of PBA notwithstanding the time of incubation. Only in the case of cultures incubated with 15 mmol/L of phenylbutyrate, we observed a significant nearly 3-fold increase in apoptosis in comparison to control cells. However, it is worth of note that the cells detected as apoptotic were mostly at the early apoptotic phase. It is interesting that PBA, at the concentration of 15 mmol/L, evoked apoptosis to a similar extent after 24 h (22.13 % ± 0.26) as well as after 48 h (22.16 ± 0.49) of incubation. It suggests that PBA is acting in strictly dose-dependent but not time-dependent manner (Fig. 4).

### The effect of phenylbutyrate on cell cycle distribution

**Fig. 5:**
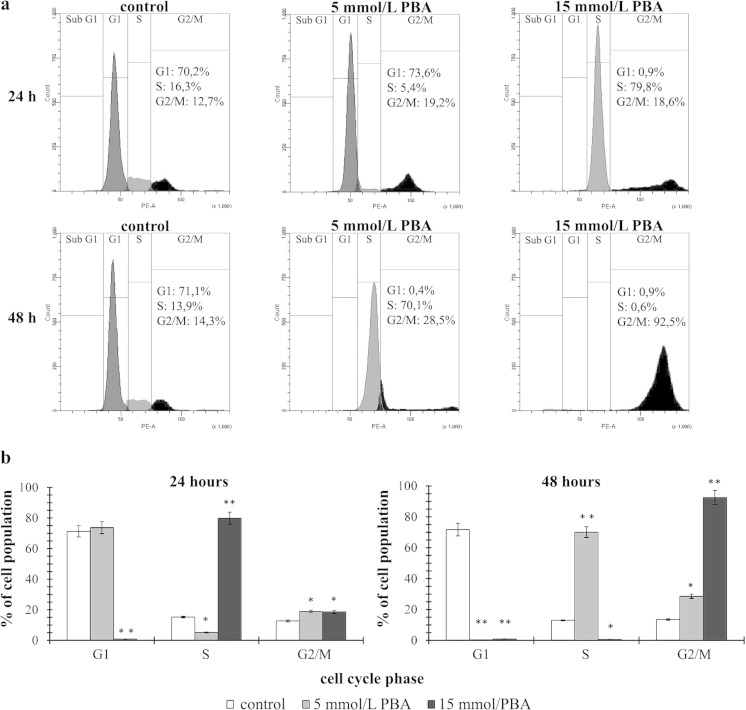
The effect of phenylbutyrate on cell cycle distribution of LN-229 glioblastoma cell line. The cell cycle was measured by propidium iodide staining followed by flow cytometry analysis. Results are shown for cells treated with 5 and 15 mmol/L PBA for 24 and 48 h versus untreated controls. **a** Graphical representation of the cell cycle profiles obtained from flow cytometry measurements. **b** Bar graph presenting the percentage of cell cycle distribution in LN-229 cells. Significant alterations are expressed relative to controls and marked with *asterisks*. Statistical significance was *p < 0.05 or **p < 0.001

To further evaluate if the proliferation-suppressive mechanism of PBA on glioblastoma cancer cells was attributable to the cell cycle arrest, we monitored the changes in the cell cycle distribution by the flow cytometry method. We observed that the cell cycle distribution of LN-229 cells was interrupted by PBA treatment (Fig. 5). The results of 24- and 48-h treatment indicated that glioblastoma cells subjected to 5 and 15 mmol/L concentrations of PBA showed different distributions of the cell cycle phases (Fig. 5a). After 24 h of incubation with 5 mmol/L of PBA, we did not observe almost any changes in the G1 phase cells; however, we noticed marked decrease in S phase cells (16.3 vs. 5.4 %) and the significant increase in G2/M phase cells (12.7 vs. 19.2 %) in comparison to the control (Fig. 5b). In contrast, 48-h treatment with 5 mmol/L of PBA resulted in complete decrease in G1 phase, with simultaneous dramatic increase in S (13.9 vs. 70.1 %) and G2/M phase cells (14.3 vs. 28.5 %) (Fig. 5b). The effects of 5 mmol/L PBA treatment seemed to be augmented in higher 15 mmol/L PBA concentration. Accordingly, after 24 h, 15 mmol/L PBA caused instantaneous depletion in G1 phase cells accompanied by the dramatic increase in S phase cells (16.3 vs. 79.8 %) and significant increase in G2/M phase (Fig. 5b), while 48-h treatment shifted practically all the cell population (92.5 %) toward G2/M phase (Fig. 5b). These results indicate that LN-229 cells did not undergo an immediate cell cycle arrest after 24 h of 5 mmol/L PBA treatment; however, 15 mmol/L of PBA accelerates the transition through the G1/S phase and enables the S/G2 progression, resulting in complete loss of cells in G1 phase. After 48 h, both concentrations evoked total depletion of G1 phase cells with a prominent increase in cells being at S- and G2/M phases. Therefore, we conclude that PBA is able to induce the cell cycle arrest in S and G2/M phases, and this effect is clearly time- and dose-dependent.

### The effect of phenylbutyrate on gene expression

**Fig. 6:**
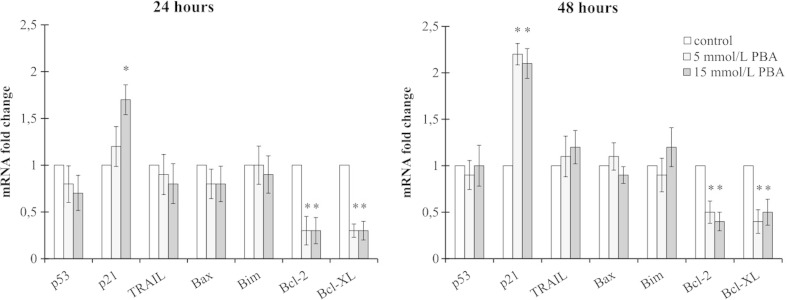
Relative quantification of gene expression in RNA extracted after 24 and 48 h from LN-229 cells treated with 5 and 15 mmol/L of PBA. Results are shown as a relative fold change in mRNA expression in comparison to control, where expression was set as 1. Statistical significance was considered if *p < 0.05

HDACI mode of action is connected with altered transcriptional regulation and changed pattern of gene expression. To investigate if the apoptotic and cell cycle altering effects of PBA were due to the changes in gene expression, we performed RT-qPCR analyses of some genes involved in cell cycle control and apoptosis. Phenylbutyrate is mostly known for evoking cellular effects in P53/P21-dependent manner. According to this, to check if the PBA-mediated cell cycle disturbances in LN-229 cells involve this molecular pathway, we measured the expression of *p53* and *p21* on the messenger RNA (mRNA) level. Interestingly, we did not observe any changes in *p53* transcript, but a significant increase in *p21* expression was noticed. After 24 h of 5 mmol/L PBA treatment, the expression level of *p21* was not significantly altered; however, incubation with 15 mmol/L of PBA resulted in significant up-regulation of *p21* (1.7-fold change in comparison to control). Forty-eight-hour treatment with both 5 and 15 mmol/L of PBA caused over 2-fold increase in the expression level of *p21* (Fig. 6). These results may suggest that PBA treatment affects LN-229 cells without engaging P53-mediated signaling. Next, we decided to identify the probable pathways involved in PBA-mediated cell death. Tumor necrosis factor-related apoptosis-inducing ligand (TRAIL) as the up-stream activator of death receptors was chosen to indicate the potential involvement of extrinsic apoptotic pathway. However, we did not see any changes in its expression. Finally, we decided to investigate if the mediators of the mitochondrial apoptotic pathway are engaged in evoking LN-229 cell apoptosis. Accordingly, we analyzed the level of *Bim*, *Bax*, *Bcl-2*, and *Bcl-X*_*L*_ expression. We did not observe significant changes in the expression of pro-apoptotic *Bim* or *Bax*. It is interesting that we did notice a significant down-regulation of the anti-apoptotic *Bcl-2* and *Bcl-X*_*L*_ mRNAs. Surprisingly, the decrease of *Bcl-2*/*Bcl-X*_*L*_ transcript levels was pronounced independently on the PBA concentration, while the flow cytometry analysis showed enhanced apoptosis only in 15 mmol/L PBA. This observation suggests that deregulation of transcription of the genes involved in mitochondrial-derived apoptosis might play an important role in regulation of PBA-dependent apoptosis in LN-229 cells. Nevertheless, the role of this mechanism cannot be undoubtedly stated.

## Discussion

Despite many researches exploring PBA influence on various cancer cell lines have already been conducted, the molecular mechanisms initiated during PBA-mediated cellular responses are still incompletely understood. The effects of PBA seem to be very cell-type-specific and sometimes limited exclusively to a particular cell line. To uncover more information about PBA activity in glioblastoma, we decided to investigate the effect of PBA treatment on LN-18 and LN-229 glioblastoma cell lines, which have not been evaluated in the context of PBA responsiveness yet. In our study, we provided novel information concerning cellular effects of phenylbutyrate treatment in glioblastoma cells.

It has been well established that HDAC inhibitors affect many aspects of cellular functioning. The primary effect of HDACIs is attributed to the alterations in the gene expression patterns caused by changes in chromatin structure. PBA has been extensively examined in various cancer cell lines showing good efficacy in triggering cell differentiation, G1 cell cycle arrest, and apoptosis [7]. Nevertheless, modest number of data exists in context of how PBA influences glioblastoma cells. There have been reports that phenylbutyrate exhibits the growth inhibitory effect in various glioblastoma cell lines mostly through the induction of cell cycle arrest, apoptosis, cell differentiation, and radiosensitization [14, 27]; however, the results of clinical trials were not as satisfactory as cell line-based studies [40].

In our study, we observed that although PBA inhibited the activity of HDACs in both cell lines to a similar extent, only LN-229 cells showed drastic dose-dependent reduction of cell viability. LN-18 cells seemed to be PBA-resistant even at high concentrations of phenylbutyrate (exceeding 10 mmol/L of PBA in culture medium). We noted marginal, about 15 %, decrease in viability of the LN-18 cells, which, although statistically relevant, did not result in any significant cellular effects in these cells (data not shown). This observation seems to be confusing since it might suggest HDAC inhibitory-independent mode of PBA action. Since PBA is class I and II HDAC inhibitor, one of our hypotheses trying to explain this phenomenon is connected with the cellular localization of class II HDACs. These enzymes may localize in the nucleus and cytoplasm, suggesting potential extranuclear functions connected with regulation of the acetylation status of non-histone proteins [41]. Acetylation of non-histone proteins has been shown to alter protein functions by modifying their stability, cellular localization, and protein–nucleotide/protein–protein interactions. Well-known targets of non-histone acetylation include essential cellular signaling mediators and transcription factors such as the following: p53, NF-κB, p65, (CREB)-binding protein (CBP), p300, STAT3, tubulin, PC4, GATA factors, nuclear receptors, c-Myc, hypoxia-inducible factor (HIF)-1α, FoxO1, heat-shock protein (Hsp)-90, cell cycle regulator-E2F, FLT3 kinase, c-Raf kinase, and many others [42]. It is noteworthy that many non-histone targets of acetylation are factors relevant for tumorigenesis, cancer cell proliferation, and immune functions. Perhaps, different pattern of protein expression or the mutation status of genes being potential targets of non-histone acetylation in LN-18 and LN-229 cells might potentially be connected with differential response to PBA treatment. Nevertheless, these assumptions must be treated with caution, and further comprehensive analyses on the transcriptomic and proteomic level are required to undoubtedly solve this issue. Our results of cell sensitivity to PBA seem to be supported by the observations of Lopez et al. who examined four glioblastoma cell lines (D54, U-251, U87-MG, and SKMG-3) and found U87-MG cells to be PBA-insensitive even after 5 days of treatment [14]. The authors have not figured out the mechanisms underlying this lack of U87-MG sensitivity to PBA. They have suggested that this might be related to the P53 status of the cells; however, this assumption fails to explain our results, since both LN-18 and LN-229 cell lines share mutated P53. This confirms highly cell-type-dependent mode of action of phenylbutyrate. According to this, we decided to conduct further experiments on LN-229 cell line only.

We noticed that the disturbances in cell viability were mirrored in diminished cell proliferation and altered cell morphology. Microscopic observations confirmed that LN-229 cells subjected to PBA treatment showed visible reduction in cell density and changes in cell phenotype. We observed prominent enlargement of cells with loss of spindle-shaped cells. This is consistent with previous studies demonstrating phenotype modulatory effect of PBA on glioblastoma, medulloblastoma, or human liver carcinoma cells [1, 43, 44]. Interestingly, obvious nuclear condensation was also visible, especially in cells treated with high concentrations of PBA. Nuclear condensation might be an indicator of cells undergoing apoptosis. Surprisingly, we did not manage to confirm any increase in apoptotic cell death in cells treated with 5 mmol/L PBA. Only 15 mmol/L PBA-treated cells showed significant increase in apoptosis ratio. The mechanisms responsible for PBA-mediated apoptotic cell death are not clear. It has been reported that in MCF7 breast cancer cells and DU145 prostate cancer cells, Fas/Fas ligand tract might be involved in apoptosis induction [45, 46], while other reports showed down-regulation of the anti-apoptotic Bcl-2/Bcl-X_L_ and overexpression of the pro-apoptotic Bax, Puma, and Noxa proteins as the potential mechanism of apoptosis initiated during PBA treatment [21, 24]. These discrepancies in phenylbutyrate-dependent modes of action between different cell lines suggest that both extrinsic or mitochondrial pathways might be involved in PBA-mediated apoptosis. TRAIL, as an upstream activator of death receptors DR4 and DR5, might indicate the involvement of extrinsic apoptotic pathway in cell death process. Our results showed no significant changes in *TRAIL* expression. This finding is in agreement with previous report of Zhang et al. who demonstrated that neither Fas/Fas ligand, tumor necrosis factor (TNF)-α, TNF-β, nor TRAIL expressions were altered by PBA treatment [17]. However, we found a prominent decrease in anti-apoptotic *Bcl-2* and *Bcl-X*_*L*_ transcripts but no changes in pro-apoptotic *Bax* and *Bim* mRNA levels, in cells treated with either 5 and 15 mmol/L PBA. Our findings support the results of previous studies conducted on prostate cancer cells demonstrating down-regulation of Bcl-2 and Bcl-X_L_ [21, 46]. However, the up-regulation of pro-apoptotic genes was not confirmed in our studies. Notably, in previous works reporting higher expressions of pro-apoptotic proteins such as Bax, analyses were performed on protein level [24, 46]. We hypothesize that this increase in expression of the apoptosis agonists might rather be the result of protein-protein interactions, since anti-apoptotic Bcl-2 and Bcl-X_L_ may bind and inactivate pro-apoptotic Bim and Bax [47]. In this respect, the down-regulation of anti-apoptotic *Bcl-2* and *Bcl-X*_*L*_ genes resulting in lower expression levels of their protein products might be sufficient to cause an increase in protein level of unbound pro-apoptotic molecules. Nevertheless, this mechanism does not profoundly explain the pattern of apoptosis induction by PBA in LN-229 cells, since marked down-regulation of *Bcl-2*/*Bcl-X*_*L*_ and no influence on *Bim* and *Bax* were observed with both (5 and 15 mmol/L) concentrations of PBA, and significant apoptotic cell death was found only in cells grown with 15 mmol/L of PBA. This suggests that more complicated pathways are involved in mediating apoptotic process in phenylbutyrate-supplemented cells, and considering only primary –gene expression modulatory effect of PBA might be insufficient to elucidate its role in cellular processes. Thus, further complex analyses on transcriptional as well as proteomic level should be done to comprehensively explain these mechanisms.

Nevertheless, apoptosis-evoking effect of PBA seems not to be the main mechanism underlying the inhibition of LN-229 cell proliferation. Since only 20 % of the cells were killed after stimulation with 15 mmol/L of PBA, it seems reasonable to have doubts if PBA treatment may be sufficient to give effective glioblastoma-suppressive effect in monotherapy. Indeed, PBA is a reversible HDAC inhibitor and normal level of histone acetylation has been proven to be restored after drug removal [14, 48, 49]. Nonetheless, it has been demonstrated that PBA shows radiosensitizing and chemosensitizing effect in glioblastoma cells [14, 50, 51]. In this respect, PBA might rather be considered as a well-tolerated non-toxic co-therapeutic cytostatic drug, continuously taken to support conventionally accepted treatments.

We further found that PBA strongly influences cell cycle distribution in LN-229 cells. Cell cycle analysis revealed that PBA caused cell cycle arrest at the S and G2/M phases accompanied by a complete loss of cells in G1. These results give a new insight into cell cycle modulatory effect of PBA, as they differ significantly from the findings presented in other cancer studies. Most of the reports infer that PBA causes strong induction of G1-dependent cell cycle arrest with prominent reduction of cells at the S stage [28]. Here, it was interesting to find that PBA evoked simultaneous shift of almost all cells to S and, finally, G2/M phases, in time- and dose-dependent manner, in LN-229 cells. Consistent with our findings, Li et al. demonstrated that SGC-7901 and MGC-803 gastric carcinoma cell lines might be arrested at the S or G2/M phases dependently on the PBA dosage [18]. In contrary, Lopez et al. observed no changes in cell cycle distribution in any of the tested glioblastoma cell lines (D54 cells, U87-MG, U251, and SKMG-3) [14]. As far as we know, no one has ever demonstrated that PBA may provoke such drastic changes in cell cycle distribution of glioblastoma cells or any other cancer cell line.

It has been assumed that the cell cycle inhibition in S and G2/M phase is mediated by the up-regulation of P53 and induction of P21 [52]. However, we did not manage to find elevated level of *p53* mRNA, while *p21* was up-regulated in PBA-treated cells. It has been known that Cdk inhibitor P21 may be induced by both P53-dependent and P53-independent mechanisms, and induction of P21 is attributable to cell cycle arrest [53]. According to our findings, PBA-mediated stimulation of *p21* expression might seem to be P53-independent, which would be consistent with the fact that LN-229 cells possess mutated P53. It is already known that eukaryotic cell cycle is controlled by the coordinated activity of protein kinase complexes, consisting of a Cdk and cyclins. Progression through G1 is regulated by the cyclin A–Cdk2 complex, entry into S phase by cyclin E–Cdk2 complex, and the G2/M phase transition is driven by cyclin B–Cdc2. It is also known that these cyclin–Cdk complexes often bind to the endogenous inhibitor proteins P21 and P27, which inhibit their kinase activities and prevent cell cycle progression [54]. P21, as a Cdk inhibitor, may also directly inhibit Cdk2, Cdk3, Cdk4, and Cdk6 activity [55]. This may indicate that S and G2/M cell cycle arrest observed in our study might be mediated by P21; however, additional experiments are still needed to provide a conclusive answer.

In current study, we provided evidence that phenylbutyrate inhibits growth and proliferation of LN-229 glioblastoma cells in time- and dose-dependent fashion and that this inhibition occurs mostly by provoking S and G2/M cell cycle arrest and apoptosis. We found that PBA was able to up-regulate the expression of *p21* however, without influencing *p53* expression, and that this up-regulation may be correlated with S and G2/M cell cycle arrest. Moreover, the anti-apoptotic genes *Bcl-2*/*Bcl-X*_*L*_ were markedly down-regulated, probably facilitating apoptotic cell death in LN-229 cells. We also found that LN-18 cells were PBA-insensitive, although possessing the same profile of main oncogenes and tumor suppressor genes as LN-229 cell line (mutated P53, deleted P14ARF and P16, and wild-type PTEN). This shows that PBA may act in clearly cell-type-specific way, and that mechanisms initiated during PBA-dependent response are variable and complex. These discrepancies in cellular effects evoked by phenylbutyrate might be the reason of unsatisfactory results of PBA treatment in patients and unsuccessful attempts to introduce PBA as a therapy for malignant gliomas in clinics. Therefore, it is worthwhile to concurrently determine all possible molecular mechanisms activated by phenylbutyrate treatment to use it successfully as a potential single therapeutic or a co-therapeutic agent for glioblastoma multiforme therapy.

## References

[1] Svechnikova I, Almqvist PM, Ekström TJ (2008). HDAC inhibitors effectively induce cell type-specific differentiation in human glioblastoma cell lines of different origin. Int J Oncol.

[2] Nagpal J, Jamoona A, Gulati ND, Mohan A, Braun A, Murali R (2006). Revisiting the role of p53 in primary and secondary glioblastomas. Anticancer Res.

[3] Koul D (2008). PTEN signaling pathways in glioblastoma. Cancer Biol Ther.

[4] Yin D, Ogawa S, Kawamata N, Tunici P, Finocchiaro G, Eoli M (2009). High-resolution genomic copy number profiling of glioblastoma multiforme by single nucleotide polymorphism DNA microarray. Mol Cancer Res.

[5] Kim TY, Zhong S, Fields CR, Kim JH, Robertson KD (2006). Epigenomic profiling reveals novel and frequent targets of aberrant DNA methylation-mediated silencing in malignant glioma. Cancer Res.

[6] Takai N, Narahara H (2010). Histone deacetylase inhibitor therapy in epithelial ovarian cancer. J Oncol.

[7] Kusaczuk M, Bartoszewicz M, Cechowska-Pasko M (2015). Phenylbutyric acid: simple structure—multiple effects. Curr Pharm Des.

[8] Pan LN, Lu J, Huang B (2007). HDAC inhibitors: a potential new category of anti-tumor agents. Cell Mol Immunol.

[9] Ropero S, Esteller M (2007). The role of histone deacetylases (HDACs) in human cancer. Mol Oncol.

[10] Koutsounas I, Giaginis C, Theocharis S (2013). Histone deacetylase inhibitors and pancreatic cancer: are there any promising clinical trials?. World J Gastroenterol.

[11] Carducci MA, Nelson JB, Chan-Tack KM, Ayyagari SR, Sweatt WH, Campbell PA (1996). Phenylbutyrate induces apoptosis in human prostate cancer and is more potent than phenylacetate. Clin Cancer Res.

[12] Steinmann J, Halldórsson S, Agerberth B, Gudmundsson GH (2009). Phenylbutyrate induces antimicrobial peptide expression. Antimicrob Agents Chemother.

[13] Zeitlin PL, Diener-West M, Rubenstein RC, Boyle MP, Lee CK, Brass-Ernst L (2002). Evidence of CFTR function in cystic fibrosis after systemic administration of 4-phenylbutyrate. Mol Ther.

[14] Lopez C, Feng FY, Herman JM, Nyati MK, Lawrence TS, Ljungman M (2007). Phenylbutyrate sensitizes human glioblastoma cells lacking wild-type p53 function to ionizing radiation. Int J Radiat Oncol Biol Phys.

[15] Wang CT, Meng M, Zhang JC, Jin CJ, Jiang JJ, Ren HS (2008). Growth inhibition and gene induction in human hepatocellular carcinoma cell exposed to sodium 4-phenylbutanoate. Chin Med J (Engl).

[16] Liu L, Hudgins WR, Miller AC, Chen L-H, Samid D (1995). Transcriptional up-regulation of TGF-alpha by phenylactate and phenylbutyrate is associated with differentiation of human melanoma cells. Cytokine.

[17] Zhang X, Wei L, Yang Y, Yu Q (2004). Sodium 4-phenylbutyrate induces apoptosis of human lung carcinoma cells through activating JNK pathway. J Cell Biochem.

[18] Li LZ, Deng HX, Lou WZ, Sun XY, Song MW, Tao J (2012). Growth inhibitory effect of 4-phenyl butyric acid on human gastric cancer cells is associated with cell cycle arrest. World J Gastroenterol.

[19] Bai LY, Omar HA, Chiu CF, Chi ZP, Hu JL, Weng JR (2011). Antitumor effects of (S)-HDAC42, a phenylbutyrate-derived histone deacetylase inhibitor, in multiple myeloma cells. Cancer Chemother Pharmacol.

[20] Kulp SK, Chen CS, Wang DS, Chen CY, Chen CS (2006). Antitumor effects of a novel phenylbutyrate-based histone deacetylase inhibitor, (S)-HDAC-42, in prostate cancer. Clin Cancer Res.

[21] Goh M, Chen F, Paulsen MT, Yeager AM, Dyer ES, Ljungman M (2001). Phenylbutyrate attenuates the expression of Bcl-X(L), DNA-PK, caveolin-1, and VEGF in prostate cancer cells. Neoplasia.

[22] Ammerpohl O, Trauzold A, Schniewind B, Griep U, Pilarsky C, Grutzmann R (2007). Complementary effects of HDAC inhibitor 4-PB on gap junction communication and cellular export mechanisms support restoration of chemosensitivity of PDAC cells. Br J Cancer.

[23] Hattori Y, Fukushima M, Maitani Y (2007). Non-viral delivery of the connexin 43 gene with histone deacetylase inhibitor to human nasopharyngeal tumor cells enhances gene expression and inhibits in vivo tumor growth. Int J Oncol.

[24] Condorelli F, Gnemmi I, Vallario A, Genazzani AA, Canonico PL (2008). Inhibitors of histone deacetylase (HDAC) restore the p53 pathway in neuroblastoma cells. Br J Pharmacol.

[25] de Conti A, Tryndyak V, Koturbash I, Heidor R, Kuroiwa-Trzmielina J, Ong TP (2013). The chemopreventive activity of the butyric acid prodrug tributyrin in experimental rat hepatocarcinogenesis is associated with p53 acetylation and activation of the p53 apoptotic signaling pathway. Carcinogenesis.

[26] Kuroiwa-Trzmielina J, de Conti A, Scolastici C, Pereira D, Horst MA, Purgatto E (2009). Chemoprevention of rat hepatocarcinogenesis with histone deacetylase inhibitors: efficacy of tributyrin, a butyric acid prodrug. Int J Cancer.

[27] Kamitani H, Taniura S, Watanabe K, Sakamoto M, Watanabe T, Eling T (2002). Histone acetylation may suppress human glioma cell proliferation when p21 WAF/Cip1 and gelsolin are induced. Neuro Oncol.

[28] Zhou Q, Dalgard CD, Wynder C, Doughty MD (2011). Histone deacetylase inhibitors SAHA and sodium butyrate block G1-to-S cell cycle progression in neurosphere formation by adult subventricular cells. Neuroscience.

[29] Wang QM, Feinman R, Kashanchi F, Houghton JM, Studzinski GP, Harrison LE (2000). Changes in E2F binding after phenylbutyrate-induced differentiation of Caco-2 colon cancer cells. Clin Cancer Res.

[30] Finzer P, Kuntzen C, Soto U, zur Hausen H, Rosl F (2001). Inhibitors of histone deacetylase arrest cell cycle and induce apoptosis in cervical carcinoma cells circumventing human papillomavirus oncogene expression. Oncogene.

[31] Carmichael J, DeGraff WG, Gazdar AF (1987). Evaluation of a tetrazolium-based semiautomated colorimetric assay: assessment of chemosensitivity testing. Cancer Res.

[32] Ho K, Yazan LS, Ismail N, Ismail M (2009). Apoptosis and cell cycle arrest of human colorectal cancer cell line HT-29 induced by vanillin. Cancer Epidemiol.

[33] Mizuno S, Bogaard HJ, Voelkel NF (2009). Umeda1 Y, Kadowaki M, Ameshima S, Miyamori I, Ishizaki T. Hypoxia regulates human lung fibroblast proliferation via p53-dependent and -independent pathways. Respir Res.

[34] Lala S, Dheda K, Chang JS, Huggett JF, Kim LU, Johnson MA (2007). The pathogen recognition sensor, NOD2, is variably expressed in patients with pulmonary tuberculosis. BMC Infect Dis.

[35] Shen JK, Du HP, Ma Q, Yang M, Wang YG, Jin J (2013). 4-Chlorobenzoyl berbamine, a novel berbamine derivative, induces apoptosis in multiple myeloma cells through the IL-6 signal transduction pathway and increases FOXO3a-Bim expression. Oncol Rep.

[36] Zhang T, Jiang B, Zou S-T, Liu F, Hua D (2015). Overexpression of B7-H3 augments anti-apoptosis of colorectal cancer cells by Jak2-STAT3. World J Gastroenterol.

[37] Hebb ALO, Moore SC, Bhan V, Robertson GS (2011). Effects of IFN-B on TRAIL and Decoy receptor expression in different immune cell populations from MS patients with distinct disease subtypes. Autoimmune Dis.

[38] Quiroz FG, Posada OM, Gallego-Perez D, Higuita-Castro N, Sarassa C, Hansford DJ (2010). Housekeeping gene stability influences the quantification of osteogenic markers during stem cell differentiation to the osteogenic lineage. Cytotechnology.

[39] Pfaffl MW (2001). A new mathematical model for relative quantification in real-time RT–PCR. Nucleic Acids Res.

[40] Camacho LH, Olson J, Tong WP, Young CW, Spriggs DR, Malkin MG (2007). Phase I dose escalation clinical trial of phenylbutyrate sodium administered twice daily to patients with advanced solid tumors. Invest New Drugs.

[41] Lucio-Eterovic AK, Cortez MA, Valera ET, Motta FJ, Queiroz RG, Machado HR (2008). Differential expression of 12 histone deacetylase (HDAC) genes in astrocytomas and normal brain tissue: class II and IV are hypoexpressed in glioblastomas. BMC Cancer.

[42] Singh BN, Zhang G, Hwa YL, Li J, Dowdy SC, Jiang SW (2010). Nonhistone protein acetylation as cancer therapy targets. Expert Rev Anticancer Ther.

[43] Li XN, Parikh S, Shu Q, Jung HL, Chow CW, Perlaky L (2004). Phenylbutyrate and phenylacetate induce differentiation and inhibit proliferation of human medulloblastoma cells. Clin Cancer Res.

[44] Meng M, Jiang JM, Liu H, In CY, Zhu JR (2005). Effects of sodium phenylbutyrate on differentiation and induction of the P21WAF1/CIP1 anti-oncogene in human liver carcinoma cell lines. Chin J Dig Dis.

[45] Chopin V, Toillon R-A, Jouy N, Le Bourhis X (2002). Sodium butyrate induces P53-independent, Fas-mediated apoptosis in MCF-7 human breast cancer cells. Br J Pharmacol.

[46] Ng AY, Bales W, Veltri RW (2000). Phenylbutyrate-induced apoptosis and differential expression of Bcl-2, Bax, p53 and Fas in human prostate cancer cell lines. Anal Quant Cytol Histol.

[47] Martinou J-C, Youle RJ (2011). Mitochondria in apoptosis: Bcl-2 family members and mitochondrial dynamics. Dev Cell.

[48] Leng Y, Chuang DM (2006). Endogenous alpha-synuclein is induced by valproic acid through histone deacetylase inhibition and participates in neuroprotection against glutamate-induced excitotoxicity. J Neurosci.

[49] Munshi A, Kurland JF, Nishikawa T, Tanaka T, Hobbs ML, Tucker SL (2005). Histone deacetylase inhibitors radiosensitize human melanoma cells by suppressing DNA repair activity. Clin Cancer Res.

[50] Lin CJ, Lee CC, Shih YL, Lin CH, Wang SH, Chen TH (2012). Inhibition of mitochondria- and endoplasmic reticulum stress-mediated autophagy augments temozolomide-induced apoptosis in glioma cells. PLoS One.

[51] Entin-Meer M, Rephaeli A, Yang X, Nudelman A, VandenBerg SR, Haas-Kogan DA (2005). Butyric acid prodrugs are histone deacetylase inhibitors that show antineoplastic activity and radiosensitizing capacity in the treatment of malignant gliomas. Mol Cancer Ther.

[52] Wolter F, Akoglu B, Clausnitzer A, Stein J (2001). Downregulation of the cyclin D1/Cdk4 complex occurs during resveratrol-induced cell cycle arrest in colon cancer cell lines. J Nutr.

[53] Gartel AL, Tyner AL (2002). The role of the cyclin-dependent kinase inhibitor p21 in apoptosis. Mol Cancer Ther.

[54] Yadav V, Sultana S, Yadav J, Saini N (2012). Gatifloxacin induces S and G2-phase cell cycle arrest in pancreatic cancer cells via p21/p27/p53. PLoSONE.

[55] Zhu H, Zhang L, Wu S, Teraishi F, Davis JJ, Jacob D (2004). Induction of S-phase arrest and p21 overexpression by a small molecule 2[[3-(2,3-dichlorophenoxy)propyl] amino]ethanol in correlation with activation of ERK. Oncogene.

